# How history of mathematics can help to face a crisis situation: the case of the polemic between Bernoulli and d’Alembert about the smallpox epidemic

**DOI:** 10.1007/s10649-021-10077-6

**Published:** 2021-07-16

**Authors:** Katalin Gosztonyi

**Affiliations:** ELTE Eötvös Loránd University, Budapest, Hungary

**Keywords:** History of mathematics, History in mathematics education, COVID-19, Collaboration with teachers, Transitional objects

## Abstract

In this article, I present the eighteenth century’s polemic of Bernoulli and d’Alembert concerning the smallpox epidemic and a prevention method called inoculation. Through an analysis of the polemic and the related resources, I show that this historical debate has various interests for mathematics education; and more specifically it can help teachers to confront dilemmas emerging with the COVID-19 pandemic (for example if a teacher should talk about it in class or not, how to help students to interpret the statistical data and the mathematical models connected to the pandemic and more generally, how to deal with the confusions and concerns emerging in connection to the pandemic). I describe the documents related to the historical polemic as transitional objects, having a potential to reveal the teachers’ own professional or personal experiences, reflections and questions, and to stimulate dialogue with them on these issues. I illustrate this proposition by the presentation of an online reading seminar realized with a French group of teachers in April 2020.

## Introduction

During the spring of 2020, when the COVID-19 pandemic exploded and was spread around the world, teachers and students had to face various difficulties related to the pandemic, including fear from the illness itself, an overwhelming quantity of information raising problems of interpretation, questions of reliability, and which were sometimes contradictory, the quickly changing and radical public measures based on that information, isolation and practical problems due to the lockdown, and the closing of schools and adaptation to online teaching.

In this situation, many teachers were confronted with the dilemma: should they talk about the pandemic in their classes or not? The question might be raised slightly differently in different countries. This is the case at least in France and in Hungary, the two countries about which the author of this paper has a closer knowledge. In France, talking about current societal issues in connection with the teaching of different disciplines is often considered important^1^. In the case of the COVID-19 epidemic, this could seem relevant on one hand because of the general fear and unease of the students, and on the other hand because of reasons related to specific disciplines. Concerning mathematics, what appeared to be particularly significant is that decisions radically influencing students’ lives were made on the basis of statistical data and mathematical models which were public and widely discussed in the media, but often difficult to understand and to interpret. This problem contributed to the confusion of students. At the same time, French colleagues^2^ related hesitations. Sometimes they themselves had difficulties to follow the quickly changing information and thus to give well-grounded answers to students’ questions. Furthermore, they found that while the topic surrounded students everywhere, its discussion in class could increase anxiety instead of easing it.

In Hungary, talking about current events in school is much less natural and usual, and can be seen as a crossing of boundaries for many teachers. So the dilemma appears a bit differently than in France. Even a teacher who feels that it is important to address the topic of the pandemic in class, because of their personal comfort or to help students interpret the overwhelming amount of information, might hesitate to feel that it is legitimate. Among ten Hungarian teachers asked about this topic^3^, all invoked the question of the pandemic with students during the spring 2020, but in most cases very briefly, mainly in informal discussions initiated by students beyond mathematics lessons, and for several teachers only before the lockdown. Only two of them claimed to work substantially on the mathematical aspects of the pandemic; and several of them raised a similar problem as French teachers, namely that students were so overwhelmed by information about COVID-19 that it did not appear desirable to come back to this subject in mathematics classes.

In both cases, a possible approach to deal with this dilemma might be to address the question indirectly, via the history of mathematics: via the discussion of historical epidemics and their management by past societies with the help of mathematical tools. The polemic between Bernoulli and d’Alembert in the eighteenth century, about statistical methods developed for the prevention of smallpox epidemics, offers a particularly interesting example.

The debate is remarkable for several reasons. First of all, the history of smallpox is interesting in itself, being the disease which first led to the elaboration of the method of vaccination and being one of the few diseases eliminated from the world thanks to vaccination. The debate in question concerns the usefulness of a method called *inoculation*, an antecedent of vaccination. Daniel Bernoulli was the first to propose a mathematical model based on statistical data with the aim of answering questions related to an epidemic. His model is an interesting example of applied mathematics (from the period of emergence of the domain of statistics), and more generally of mathematical modelling. D’Alembert’s criticism, disputing the applicability of Bernoulli’s method on the decisions about inoculation, raises interesting questions concerning scientific methodology, but also moral and political issues, including the limits of the applicability of mathematics in social problems. It is quite easy to discover the echo of these questions and considerations in the current discussions about the COVID-19 pandemic.

In this paper, I will take into consideration the different possible interests of this historical polemic for mathematics education, by making reference to generally cited reasons for including history of mathematics in mathematics education (Arcavi & Tzanakis, 2000; Fried, 2007; Jankvist, 2009). However, instead of proposing specific classroom implementations, I will focus on how the study of this historical debate can support teachers’ reflections and help them to confront efficiently the personal and professional challenges raised by the pandemic. I will base this reflection on an idea developed by Bernard and Gosztonyi (2018)—in line with didactical approaches on the teachers’ interactions with resources (Gueudet et al., 2012; Remillard, 2005)—that historical texts can be seen as *transitional objects* or in other terms *mirrors* of the teachers’ personal and professional experiences: that the reading of historical texts can stimulate dialogue with teachers about problems they are concerned with.

The polemic between Bernoulli and d’Alembert was the topic of an online reading session with a group of French teachers in April 2020. This group, existing in the frame of the Institut de Recherche sur l’Enseignement des Mathématiques (IREM) Paris Nord, was created in the spirit described above. Its members are used to reading historical texts which are then the basis for designing teaching projects, in-service teacher training sessions and resources. In March and April 2020, it was a shared desire of the members of the group to reunite around a topic related indirectly to current events, both for our own pleasure and for thinking together about the issues mentioned above.

I start the article by summarizing the content of the historical debate between Bernoulli and d’Alembert. In the second part, I first analyze various potential interests of this polemic for mathematics education in general. Then, I focus on its potential connections to the current situation, and develop the notion of *transitional object* in order to explain how these historical resources can generate and support discussions with teachers on the subject. Finally, I illustrate this proposition by presenting elements from the discussion of the reading session of the IREM group.

## Bernoulli, d’Alembert and the smallpox

### The problem: the smallpox epidemic and the inoculation

Smallpox was one of the most severe infectious diseases in human history. In the seventeenth and eighteenth centuries, smallpox epidemics were considered a major problem of European societies. It was one of the leading causes of mortality in this period, being highly infectious and with a high mortality rate (varying between 1/4 and 1/18 according to territories and periods). It particularly affected children (as the majority of the population caught smallpox before attaining adulthood). Even when it was not fatal, it often left heavy scarring or permanent blindness. However, smallpox could only be caught once: after surviving smallpox, people became immune for the rest of their life. Based on this property of the disease, a technique of prevention called *inoculation* was developed in Medieval China and practiced across Asia. Inoculation consisted of the introduction of material from smallpox pustules (and thus a weaker version of the virus) into the skin. This procedure generally caused a less severe illness than the naturally acquired smallpox and immunized the subjects of inoculation for the rest of their lives. Inoculation was not however without risks, and could lead to the patient’s death. Thus, inoculation meant to submit someone (typically children) to a small but imminent risk of death in order to gain a life-long protection against a fatal disease (Colombo & Diamanti, 2015; Gabriel & de la Harpe, 2010; Rohrbasser, 2011).

Inoculation was introduced in England in 1718 by Lady Montagu, wife of the British ambassador to the Ottoman Empire. In France, however, the method was regarded with suspicion. Various French intellectuals, including Voltaire and Diderot among others, engaged in the promotion of inoculation which was the object of debates in the second half of the eighteenth century in newspapers and at the Academy of Sciences, and became one of the important topics of the Enlightenment period. In a lecture presented at the Academy in 1754, La Condamine insisted that inoculation should be publicly promoted and suggested that the question should be seen as a mathematical problem: the decision about inoculation should be based on the comparison of risks (Colombo & Diamanti, 2015; Rohrbasser, 2011; Seth, 2014).

### Bernoulli’s proposal

It was the Swiss mathematician Daniel Bernoulli (1700–1782) who came up with a mathematical model capable of answering La Condamine’s suggestion. Following Maupertuis’ encouragement to study the inoculation problem from a mathematical point of view, he presented a study in 1760 at the Academy of Sciences, later published under the title *Essai d’une nouvelle analyse de la mortalité causée par la petite vérole, & des avantages de l’inoculation pour la prévenir* (Bernoulli, 1766)^4^.

The problem Bernoulli examined was to decide if the government should implement the inoculation of all individuals. His method consisted of the construction of a population model that allowed the comparison of two possible states of the world: one where everybody is submitted to the risk of smallpox and another where everybody is inoculated at birth. More precisely, he calculated life expectancy and compared it in the two cases when inoculation would be applied or not applied.

One of the difficulties he had to face was the availability of convenient data. Mortality tables had been published since the seventeenth century: the first, published in London in 1662, is usually considered one of the founding texts of both statistics and demography. These tables were based on bulletins reporting burials and baptisms, and were mainly used to inform people on the recurrent epidemics of plagues. Therefore, the cause of death was usually indicated but not the age at which people died. One mortality table used regularly as a reference in the eighteenth century was the one published by Halley in 1693 based on the data of Breslau. The population of this city of the Habsburg Empire was quite stable and thus the table constructed on these data could be considered “an estimate of the degrees of mortality of mankind”^5^: starting with 1000 one-year-old children, the table shows how many people of this population will be alive after each year. The table was regularly used in Europe for the calculation of probabilities to live up to a certain age, for example to compute the price of annuities (Bacaër, 2011).

Halley’s table was the mortality table that Bernoulli used for his model^6^. He established the following hypotheses:

- Smallpox can be caught once (the individuals surviving it are immunized for the rest of their life).
- Each individual (who has not caught smallpox before) has the probability called 1/*n* of being infected each year.
- The individuals infected with smallpox die from it with a probability called 1/*m* (and survive with a probability of 1-1/*m*).

Bernoulli acknowledges that the second and the third hypotheses, namely that these probabilities do not depend on age, are simplifications, but he considers that the model reasonably approaches reality. An additional issue with the third hypothesis was that it was not possible to estimate the chance of dying from smallpox depending on the age of the victims, as the age of the dead was not recorded. Bernoulli takes *m* = 8, a number which corresponds to the observations of the period. As for the second hypothesis, he explains that although at first glance the chance to be infected seems to vary highly by age, this can be explained by the fact that most people already got over smallpox in their childhood and are therefore immune when they attain adulthood. He shows that with a choice of *n* = 8, the result will be that about 1/13 of the entire population dies because of smallpox, a proportion which had been observed in several European cities (Bacaër, 2011; Bernoulli, 1766; Rohrbasser, 2011).

Bernoulli then deduces the function *s* expressing the number of people who have not been infected by smallpox at age *x*, while there are altogether ξ survivors at age *x*:

$$ s=\frac{m}{\left(m-1\right){e}^{\frac{x}{n}}+1}\xi $$

**Fig. 1:**
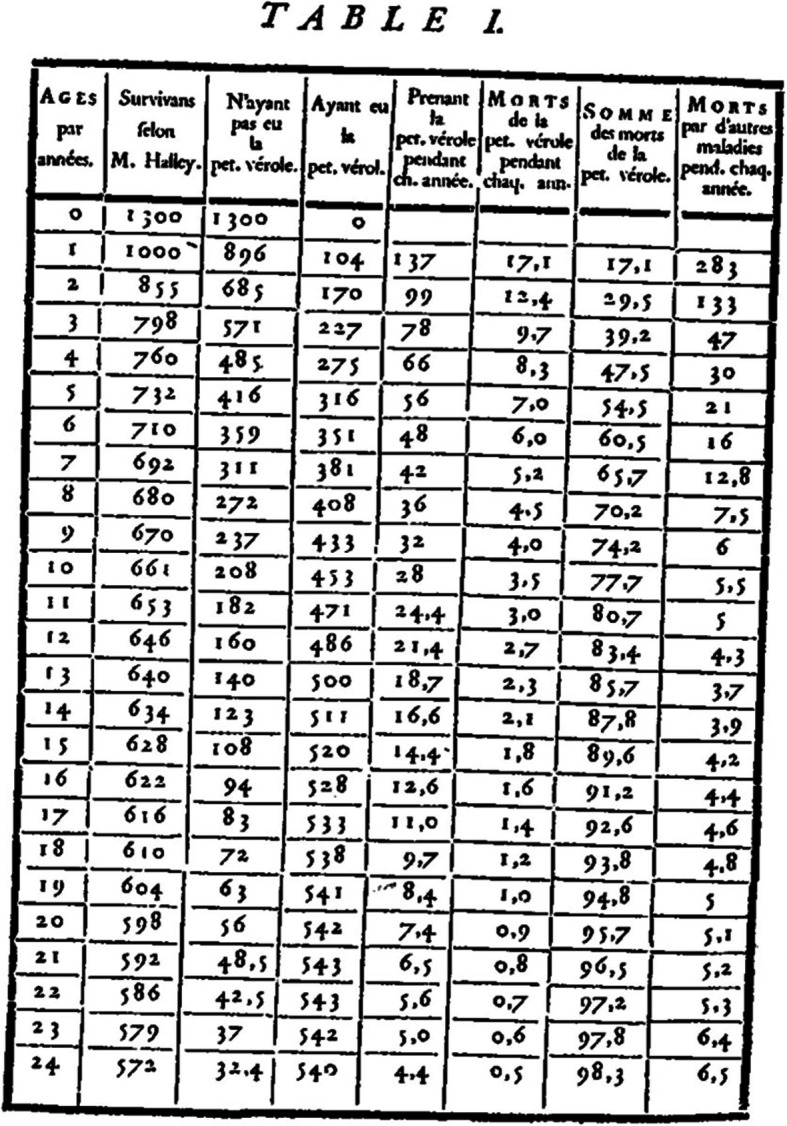
Bernoulli’s table (Bernoulli, 1766, p. 44)

Based on these calculations, Bernoulli can complete Halley’s table with new columns giving respectively the number of people (Fig. 1):

- Who have not been infected before age *x*
- Who have been infected and survived smallpox, and thus are immune at age *x*
- Infected by smallpox at age *x*
- Dying by smallpox at age *x*
- The total number of victims of the smallpox up to age *x*
- The number of people dying for other causes at the age *x*^7^

Bernoulli then prepares an alternative table where the danger of smallpox is eliminated. A calculation of life expectancy gives that with the danger of smallpox a person at birth can have a life expectancy of 26 years and 7 months while without smallpox life expectancy would be 29 years and 8 months. Thus, inoculation at birth could increase life expectancy by more than 3 years.

The danger of dying from inoculation is easy to take into account in this model: as the risk of inoculation appears in the two months following the procedure, Bernoulli simply suggests to decrease the starting number of the population by the number of the victims of inoculation and to use the second table for the rest of the counting (the rest of the population being immunized against smallpox). The risk of inoculation was difficult to estimate, but Bernoulli reasonably supposed that it was less than 1% and with this proportion the inoculation remains highly advantageous in regard to life expectancy (Bacaër, 2011; Rohrbasser, 2011). Bernoulli thus concludes:

I simply wish that, in a matter which so closely concerns the wellbeing of the human race, no decision shall be made without all the knowledge which a little analysis and calculation can provide.^8^

### D’Alembert’s criticism

D’Alembert presents his critical answer to Bernoulli’s communication at the Académie des Sciences on the 12 November 1760. The transcription of his lecture is published in 1761 as *Onzième Mémoire* in his *Opuscules mathématiques* (d’Alembert, 1761)^9^. Thus, d’Alembert’s criticism appeared several years before Bernoulli’s original work could have been published, at which Bernoulli took offense, and considered it as profoundly unfair. The reading of d’Alembert’s polemic text, going through a series of criticisms about Bernoulli’s work to arrive finally to the same conclusion as his adversary, namely that inoculation should be promoted, can indeed make a first impression of rivalry instead of a real scientific controversy. There are however thoughtful elements of his criticism, worth close examination (Colombo & Diamanti, 2015; Rohrbasser, 2011; Seth, 2014).

One major point of d’Alembert’s criticism concerns the hypothesis that the probabilities of being infected and of dying of smallpox are independent of age. D’Alembert insists that the variation of these probabilities according to the age of people should be taken into account—he even suggests an alternative model with an alternative function taking into account this variation. He claims that lacking these data, it is not reasonable to construct mathematical models on the subject.

Another main element of his criticism questions the relevance of average life expectancy as a good criterion of decision in favour or against inoculation. He claims that the calculation of life expectancy does not discriminate between different years of life and ignores that a smaller gain in young ages might be preferred to a larger gain in advanced age. In his interpretation, the 3 or 4 years that someone can earn by inoculation should be counted at the end of the given person’s life, and a young person, for example in his/her 30s, which is the most productive and enjoyable period of life according to d’Alembert, might not want to take a risk of dying immediately by inoculation for a gain realized many years later.^10^

D’Alembert does not seem to consider that what is at stake here is an average of length of life. His argument reveals however several interesting dilemmas concerning Bernoulli’s method. On one hand, it suggests that decisions are subjective when a short-term risk has to be compared to a long-term one. On the other hand, it underlines the difference between individual and collective decisions.

Bernoulli’s problem concerns the collective level, the choices of governments, where the general growth of life expectancy can be considered a gain for the state: in an era when the population was considered to be an indicator of the country’s wealth (Seth, 2014, p. 746), this could appear as a crucial contribution to the country’s well-being. But d’Alembert reminds his public that inoculation is an individual choice and individuals might have other preferences.^11^

He evokes the dilemma of the father who has to decide if he subjects his son to inoculation, with the hope of protecting him against smallpox but risking losing him in the following weeks; or if he avoids this risk and accepts that natural smallpox may take away the child some years later. Even if statistics is in favour of inoculation, it is not a consolation for a father who actively contributed to the death of his child through inoculating him.^12^ As Seth (2014) underlines, this argument is related to more general moral dilemmas of the period concerning preventative medicine:

In the debate regarding whether or not to practice this dangerous method, lobbies and individuals raised different questions: could one usurp God’s right to decide who should become ill? If one had one’s child inoculated and he or she died as a result, could one be considered to be his or her murderer? Was it not best to let nature take its course? (pp. 740–741)

In this sense, while a death by natural smallpox would be considered (passive) acceptance of God’s (or Nature’s) will, inoculation is an active engagement and one can feel responsible for his or her own death or for the death of loved ones, even if this happens with a smaller probability than death by natural smallpox.

D’Alembert underlines that he is not against inoculation; he only thinks that mathematical calculations alone are insufficient to answer the problem. Opposed to La Condamine and Bernoulli who suggest to see the problem as a comparative calculation of risks, d’Alembert claims that this kind of decision problem implying life and death is rather moral than mathematical, and too complex to be reduced to mere calculations.

### The reception of Bernoulli’s and d’Alembert’s debate

The problem of inoculation was solved with Jenner’s discovery, published in 1798. Based on the observation that milkmaids were generally immune to smallpox, Jenner made successful experiments of infecting people with cowpox in order to protect them against smallpox. The method is effective because the cowpox virus is close to that of the smallpox and thus a cowpox infection immunizes against smallpox without causing a dangerous illness in humans. From the Latin name of cowpox (*vaccinus*), the procedure was named *vaccination*, and the method, eliminating the risks of inoculation, soon was spread around the world.

Nevertheless, Bernoulli’s and d’Alembert’s polemics kept stimulating interest. The problem and the calculations were reconsidered by Lambert back in 1772 and by Duvillard in 1806 (Bacaër, 2011). Laplace gave a short and transparent summary of the debate in his *Théorie analytique des probabilités* (Laplace de Marquis, 1812). Bernoulli’s method and the polemics with d’Alembert are revisited by various recent articles in different disciplines, as in the history of medicine (Messerli et al., 2013), the history of mathematics (Daston, 1995; Gabriel & de la Harpe, 2010), population dynamics (Bacaër, 2011) or philosophy and literature (Fressoz, 2010; Seth, 2014).

## Interest of the polemic for mathematics education and connections with the current COVID-19 pandemic: theoretical considerations

### Revisiting arguments for using history in mathematics education

The debate between Bernoulli and d’Alembert can be interesting for mathematics education for various reasons, even independently of the COVID-19 pandemic. Indeed, among the numerous arguments for integrating history in mathematics education listed in overviews about the topic, several can be relevant in this case. From the five big domains of arguments listed by Arcavi and Tzanakis (2000) in the ICMI Study *History in mathematics education*, arguments related to at least three seem pertinent to this case: *a) The learning of mathematics*, *d) The affective predisposition towards mathematics* and *e) The appreciation of mathematics as a cultural endeavour*. Jankvist (2009) in his widely cited article suggests a categorization of the *whys* of using history into two main groups, seeing history as a *tool* and as a *goal* in itself. Both kinds of arguments can be underlined in our case.

Firstly, the debate can be relevant as a support for the teaching of specific mathematical domains. Bernoulli’s model can serve as an interesting example of the construction of a statistical model, namely for the calculation of life expectancy. D’Alembert’s criticism offers an early example of a subjective approach to probability (Colombo & Diamanti, 2015; Rohrbasser, 2011), and the reading of his arguments can stimulate a discussion about the different interpretations of probability.

In a more general sense, the debate provides an interesting example of mathematical modelling and of applied mathematics. The usefulness of mathematics as tool reacting to relevant problems of society is discussed in the debate, in connection with other domains of life and culture. The problem of inoculation is a good example of an open problem, where mathematics is a tool in the solution, but various kind of questions (medical, political, moral) can be considered and the solution depends on various choices and priorities. Difficulties of applied mathematics related to the availability of data are also clearly exposed in the Bernoulli-d’Alembert debate: the case might help to understand how the efficiency, applicability of statistical (and more generally mathematical) models is related to the quality of accessible data.

Furthermore, the debate about inoculation can serve as a basis for interdisciplinary discussions between mathematics and history, biology and medicine. The subject can be also relevant for the purposes of civic education. Understanding the impact of epidemics on past societies, learning about the emergence of vaccination and understanding how science could help to estimate the risks and advantages of a primitive antecedent of vaccination can engage students in a discussion about vaccination in modern societies and arm them against scientifically ungrounded propaganda. At the same time, d’Alembert’s arguments can open discussions about the limits of the applications of mathematics.

Finally, from a *history as a goal* perspective, the polemic about inoculation, connecting medical, philosophical, moral, political and mathematical considerations, can illustrate how mathematics develops in connection with the general development of societies: its development can be inspired by societies’ problems and, conversely, offers tools for confronting them.

### Historical texts as *transitional objects* for teachers, regarding the COVID-19 pandemic

The arguments listed above give general reasons for exploring the Bernoulli-d’Alembert polemic in mathematics education, independently of the context of the COVID-19 crisis. Here I examine more closely why this topic can be particularly interesting regarding the current COVID-19 pandemic. Although this approach might be relevant both to implement in the classroom and to work with teachers, I focus here on this second aspect, namely how the reading of these historical texts can support the teachers’ reflections.

The last argument given in the preceding section can be reformulated with regard to the challenge of facing the pandemic: the history of inoculation and the polemic between Bernoulli and d’Alembert help with realizing that similar crises existed in the past and are part of human history, that mathematics can contribute to facing this kind of crisis, and that crises are also motivating for the development of sciences.

In a more general sense, turning towards history of mathematics can be understood as taking a step back from the unpredictable and quickly changing present, getting some perspective by traveling to the past, to a world different from ours in many aspects. Through the reading of historical texts (or another form of exploration of a historical topic), in this case the Bernoulli-d’Alembert debate, one is free to formulate his/her own questions and reflections. Reading about the debate can help to change points of view: we take distance from the problems of the present while turning to the problems of the past, but the analogies of the historical example allow connecting freely to the issues of concern to the participants. The mix of familiarity and strangeness, and the open nature of the activity can help formulating the readers’ own reflections, and this might lead to a better understanding of the current crisis situation.

These considerations are close to what Fried (2007) proposes concerning the relevance of a historical approach in mathematics education. He suggests that examining history of mathematics, by the distance of time and the differences between ancient and contemporary mathematics, can raise awareness about the differences and thus help to better understand ourselves “as mathematical beings”.

[…] a non-perfunctory look at history can lead us from our own approach to mathematics viewed as if it were the only approach – a synchronous view in the full sense – to a view of our approach as one of many and, therefore, one *distinctly* ours. This moving full circle from our own mathematics, through history, and then back to our own mathematics becomes, accordingly, a movement towards self-knowledge, a knowledge of ourselves as a kind of creature who does mathematics, a kind of mathematical being. So, I would propose that this be the commitment of mathematics education: *our self-knowledge as mathematical beings*. (Fried, 2007)

In Bernard and Gosztonyi (2018), in the context of in-service teacher education and teachers’ professional development, we suggested that historical texts can be seen as *transitional objects.* The term *transitional object*, developed by Winnicott in child psychology, was adapted to a context of professional education (in medicine) by Bonah and Danet (2014). They use historical sanitary films in their educational program in order to provoke *astonishment* in their medical students by the temporal and/or spatial distance between the historical document and the contemporary context; and by this astonishment, initiate discussions about professional practice. The film plays the role of a *mirror*: it helps to take a step back from situations which are familiar for the viewer and put them into perspective. Furthermore, as Bonah and Danet underline, an object, such as a film (or a text), is easier to dispute and criticize than the professor’s word which has authority: Bonah and Danet also talk about the transitional character of their educational objects in this sense.

The idea of taking distance from our own context and experiences with the help of historical documents is quite similar to Fried’s approach cited above. The main difference is that, while Fried emphasizes a diachronic comparison between different eras of doing mathematics, Bonah and Danet’s approach originates in psychology: in Winnicott’s original theory, a transitional object is an intermediate between the subjective and objective reality of a child. They can thus more easily take into account the diversity of personal experiences and interests of participants.

We adopted the term *transitional object* to describe an approach elaborated for teachers’ professional development, where the historical mathematical texts are used to reveal or *mirror* the teachers’ (often implicit) own professional or personal experience, and to stimulate dialogue with them on these issues. In an earlier paper (Bernard & Gosztonyi, 2014), we described this practice as a creation of a milieu favouring an *encounter* of the experience of reading the text and the teachers’ preliminary professional and personal experience. The IREM group, presented in details below, was created in this spirit.

Of course, the choice of the texts depends on preliminary assumptions concerning teachers’ potential interest. This is based on an a priori analysis, often related to our historical or didactical research activities. We introduced the term *pre-transitional object* to characterize texts where a potential to become transitional object in an interaction with teachers was identified. The first texts we used in this sense were studied in the frame of the interdisciplinary research project entitled “Series of problems at the crossroad of cultures” (Bernard, 2015): we have shown in Bernard and Gosztonyi (2018) how the reading sessions originally planned to provoke discussions related to problem-based teaching could generate various encounters with the texts, depending on the participants’ preliminary experiences and problems.

In the current case, the documents related to the Bernoulli-d’Alembert polemic have a strong potential to play the role of transitional objects: to help confront experiences, worries and questions related to the COVID-19 pandemic. For teachers, this potential is present both on the personal and the professional level: for the reflexion of their own personal experiences and for the teaching dilemmas evoked in the introduction.

The source of this potential is the analogy of the historical pandemic and the historical debate with the current pandemic and debates. Echoes between the historical and the modern polemics can be identified on several points. One of these points concerns the difficulties of the construction of a statistical model, including the choice and the availability of convenient data, and the problems of reliability of the mathematical model. Another connection can be discovered in the confrontation of individual and collective choices underlined by d’Alembert: the conflicts between individual preferences and official measures concerning public health, and the legitimacy of mathematical models as supports for these choices. These points of connection were explored and developed during the reading session of the IREM group that I will present in the following section.

## Discussion of the debate with a group of French teachers

### The IREM group

The French IREM system is a network of institutions allowing teachers and researchers (in mathematics, mathematics education, history of mathematics, etc.) to meet and work together regularly in thematic working groups, create resources and organize in-service teacher education sessions for other teachers. The system is mainly focused on mathematics education, but some interdisciplinary groups also exist in these frames.

The group, of which I am one of the founding members, exists in the frame of the IREM Paris Nord. It focuses on historical texts and develops teaching experiences, resources and in-service teacher education on the basis of these texts. Founded in 2014, originally as an extension of an in-service teacher training related to the above mentioned “Series of problems” research project (Bernard & Gosztonyi, 2014), the organizing principle was to create a space where participants of the training session can extend their experience of *encounter* with the texts, in the sense described above. Since then, the activities of the group covered the preparation of teaching sessions for secondary school, for pre- and in-service teacher education, reflection about participants’ teaching practices, the development of research articles as well as resources for teaching and, last but not least, collective reading sessions for the pleasure of the participants. The topics of the discussions diversified during the years, beyond the “Series of problems” thematic, including mathematical recreations and education for citizenship.

It is important to underline that the relation between teachers and researchers in the group is a partnership with mutual benefits for each participant. The researchers are also teacher educators and thus their teaching practices are also discussed. Furthermore, research activities of the researcher participants are often nourished by the group discussions.

The group has two researcher members (in history of mathematics and in mathematics education), and three to six teacher members, most of them being secondary school mathematics teachers but also one history and one French and ancient language and literature teacher. This last circumstance allows us to develop interdisciplinary activities, a potential which is quite often exploited in our projects.

One of the interdisciplinary activities to which the group contributes is an in-service training session addressed to mathematics and to history teachers, focusing on mathematics, history and civic education. The subject of the Bernoulli-d’Alembert polemic about smallpox inoculation was first mentioned, prior to the COVID-19 crisis, in connection to this teacher training (without being implemented yet), as one of the first examples of using probabilities in relation with demographic and social topics, and also in connection with the increase of scepticism about sciences in society and among students, including the modern debates about vaccination.

Thus, it was a quite natural idea to come back to this topic during the lockdown due to the COVID-19 pandemic, while the theme gained a new relevance. I add at this point that the idea of working at a distance was not completely new to the group either: while I was present in Paris in the year of the group’s creation, I moved to Budapest (Hungary) since then, and in most of the cases I participate in the working sessions via video conference.

### The reading session about the Bernoulli-d’Alembert polemic

In March 2020, the idea of an online meeting, suggested by the leader of the group, was received with enthusiasm by the members. It offered a perspective of *recreation* in various senses: to escape from the worries of the pandemic, of the lockdown and of the newly established online teaching, by finding the familiar and pleasant activity of an IREM meeting, and by discovering an unknown episode of history. At the same time, among several suggested topics, the Bernoulli-d’Alembert polemic was chosen unanimously by the participants, showing a need to keep indirect connections to the COVID-19 crisis which dominated public and private discussions in this period.

Prior to the reading session, various connected resources were found and read by the participants. The subject was approached through several secondary sources, including Rohrbasser (2011), cited above, and Fressoz (2010), an article revisiting the polemic in the context of the H1N1 pandemic of 2009; and also primary sources, including Bernoulli’s and d’Alembert’s works and the short and well-accessible summary of Laplace (1812) about the debate. During the session, after a discussion of the different preliminary readings made by each participant, a part of d’Alembert’s *Onzième mémoire* (1761) was read together.

Part of the session was devoted to the discussion of Bernoulli’s method. It was surprising to discover the difficulties related to the choice and interpretation of data including the problems of availability of convenient life tables and the estimation of infection and mortality rates. Bernoulli’s struggle is visible both in his own text and in secondary literature, for example through the long discussion of the chosen parameters. This observation had strong echoes with the problems of the availability and reliability of data experienced in the spring of 2020 concerning the COVID-19 infections, where the construction of reliable mathematical models and predictions about the progression of the pandemic with the help of these models were crucial prerequisites of efficient public decisions; but these models were unstable and the reliability of predictions was limited due to missing data and information about the nature of the disease.

The mathematical complexity of Bernoulli’s model also stimulated discussion. His mathematical calculations are accessible for an average mathematics teacher but their understanding needs considerable effort; and the fact that an early model of life expectancy is of this complexity helps to acknowledge the complexity of modern mathematical models about pandemics. This question is also connected to the difficulties of interpreting data and models, presented in the form of tables, graphics, etc. in the media, that teachers as well as their students had to deal with.

In addition, the problem of the comparison of risks evoked modern discussions about risks and benefits of treatments and vaccines, a question widely discussed in connection with the COVID-19 pandemic and the various medical experiments currently conducted, while the length of time to find reliable solutions is counted.

The reading of d’Alembert’s lecture surprised us first of all by the literary and rhetoric qualities of the text. It appeared to be not only accessible but also quite pleasant reading. The introduction allows modern readers to place themselves in the context of the debate within the eighteenth century Academy of Sciences, but also gives an impression of familiarity when d’Alembert acknowledges treating a subject which might be considered tedious by the audience due to exhausting public debates about it.^13^

Understanding d’Alembert’s arguments, especially those connected to the interpretation of life expectancy appeared to be not always easy, and led to discussions about the difficulties of interpretation of probability and of statistical constructs.

One of the main questions stimulating interest during the discussion was, however, the conflict between individual and collective choices. Indeed, the problems raised by d’Alembert, described above, strongly echoed the similar conflicts experienced during the COVID-19 pandemic: there was a lot of confusion in public discussions about the role of the different protective measures, for example the wearing of masks, namely if they were meant to protect individuals or the community around them. While official measures, restrictions were made with regard to public health, their respect depended highly on individual decisions. The lockdown, the limited accessibility of public health services, etc., created problems which could be lived larger on the individual level than the threat of the disease itself. The discussion about the Bernoulli-d’Alembert polemic helped to increase consciousness concerning this ambiguity and allowed conversation about the related problems.

An unexpected Hungarian connection was also discovered during the discussion. Jelitai (1936/2018, 1937/2019) presents the diaries and the correspondence of two Teleki earls, József and Sámuel who both studied in Basel under Bernoulli in 1759–1760, and were later in contact with the French intellectual milieu in Paris. József, who had become close friend of Bernoulli, was at the Academy of Sciences when d’Alembert presented his criticism, and was obviously the one who informed Bernoulli about it. Beyond the interest of introducing a Hungarian piece in the French culture–based discussion, the elements of these diaries also helped to feel more familiar with the context: the Telekis describe not only the content of Bernoulli’s courses in Basel or the list of the other lectures presented at the Academy on the day of d’Alembert’s lecture, but also the meeting with other famous intellectuals of the period, their meals, amusements, families and lovers. It was even more interesting for the IREM group, as the work of some other characters mentioned in the diaries has been studied by the group earlier^14^. The Hungarian diaries thus helped to connect the new readings to the earlier ones. This is a good and simple example of texts having a potential to serve as transitional objects in a given context: having a historical document which connects Hungarian and French history of science is stimulating for a French working group having a Hungarian participant; and the connection created by these diaries between the former and the new readings of the group reinforce continuity in the groups’ activities.

Some weeks after the session, the participants answered a questionnaire containing the following questions:

- What was interesting, inspiring for you in the texts read and in the discussion?
- What did the reading session bring you in connection with the epidemic that we are experiencing at this moment?
- What did it bring you in terms of teaching: did it reflect ideas or problems of teaching for you (in connection with the COVID-19 or independently of it)?

The answers to this questionnaire contribute to the understanding of the participants’ motivations and interest in the session, and illustrate how the texts worked as transitional objects for them.

The idea of taking some distance from the current situation and changing the point of view clearly appears: one participant mentioned that the session offered him “an intellectual oxygen bubble”, while another described a “stepping back and getting some perspective in this period during which we were bombarded with scientific articles or pretending to be such, scientific quarrels, contradictory and more or less well argued opinions”. The pleasure is also a recurrent element of the discussion about the motivations.

In their answers, the teachers emphasized different aspects of the discussion, reflecting their personal and professional interests. One mathematics teacher underlined his interest in the example of mathematical modelling and of applied mathematics, a subject which is important for him in mathematics education; another stressed first of all her interest in the historical contextualization, which is a subject of personal curiosity for her, and also something that she used to include in her teaching activities. The history teacher rather emphasized her interest in the history of epidemics and vaccination as part of human history, and the possibility that it offers to include history of sciences in history lessons. Questions related to the analysis and interpretation of data, statistics and graphics were evoked. Finally, the question of the conflict between individual and collective choices was also recalled by participants.

Although the original motivations for the reading session were clearly connected to the pandemic, several elements of interest mentioned by the participants in the a posteriori questionnaire are described as having a general educational interest, independently from the pandemic. Other educational aspects (especially the two last one listed above, concerning the interpretation of data and the conflict between individual and collective choices) appear though in explicit connection with the COVID-19 crisis and the problems of dealing with the questions and the anxiety of students.

## Conclusion

In this article, I discussed the potentialities offered by history of mathematics to help teachers and students facing the current crisis situation due to the COVID-19 pandemic. I focused especially on the teachers’ perspective. I presented the example of the polemic between Bernoulli and d’Alembert on the subject of smallpox inoculation, as a candidate for a historical topic which can be relevant for this purpose. I analyzed the potential interests of this polemic for mathematics education which go beyond the current crisis: I insisted that the topic can support the learning of specific mathematical topics, the understanding of mathematical modelling processes and the vision of mathematics as part of human culture and as a tool to answer social crises, including pandemics.

More specifically concerning the context of the pandemic, I suggested that the discussion of the historical polemic about the smallpox pandemic can offer a potential solution to the dilemma presented in the introduction of this article: it can allow participants to raise their personal questions and reflections, in connection with mathematics and beyond, while making analogies between the historical and current debates but without forcing them to treat directly the problems and data of the current pandemic.

Nevertheless, instead of proposing direct classroom implementations of the topic, I proposed to consider historical texts as *transitional objects* in the interaction with teachers, indirectly stimulating discussions about the problems with which they are concerned. For teachers, the collective reading of the documents related to Bernoulli’s and d’Alembert’s polemic can stimulate personal and professional reflections, including general reflections of teaching mathematics, history or other subjects, reflections on the pandemic itself and reflections related to the specific educational problems generated by the pandemic. This proposition was illustrated in the last part of the article through the case of the online reading session of the French IREM group.

This kind of discussion with teachers can potentially lead to a direct implementation, in one form or another, of the historical topic in classrooms; but it can also influence indirectly the teachers’ reflections on their teaching activities and practices. In my knowledge, there has been no experience realized in classrooms on this subject, although several colleagues mentioned the planning of similar experiences in the future. The short time since the explosion of the COVID-19 pandemic and the complicated and dynamically changing circumstances did not allow collecting extensive empirical data on how exactly the reading session influenced the teachers’ work on a shorter or longer term, or on how teachers from different contexts (for example from other countries or teachers who have no preliminary experience of working with historical texts) would react to a similar discussion of the historical polemic.

In his famous article discussing the difficulties of using history in mathematics education, Siu (2007) concludes with the following claim: “No, I don’t use history of mathematics in my class. I let it permeate my class.” (p. 10). The Bernoulli-d’Alembert polemic may or may not be treated explicitly in classrooms. It is however obvious that the collective reading and the discussion of the polemic helped teachers to face the COVID-19 crisis and the problems that it raised for their teaching practice, and, with Siu’s words, it most likely “permeated their classes”.

## Footnotes

1 Resources for teachers were disseminated on the website of the ministry of education, even if quite briefly, and mainly in history and geography. See, e. g., https://eduscol.education.fr/1812/accueil-de-tous-les-eleves-l-ecole-et-au-college ; https://eduscol.education.fr/histoire-geographie/actualites/actualites.html?tx_ttnews%5btt_news%5d=8559 ; https://eduscol.education.fr/histoire-geographie/actualites/actualites/article/pandemie-mondialisation-et-frontieres.html ; https://eduscol.education.fr/histoire-geographie/actualites/actualites/article/le-covid-19-face-a-lhistoire.html

2 Members of the group History and Epistemology of the IREM (Institut de Recherche sur l’Enseignement des Mathématiques) Paris Nord. For details, see below.

3 The Hungarian teachers in question were only asked if they invoked the pandemic with students or not, without deeper interviews.

4 *An attempt at a new analysis of the mortality caused by smallpox and of the advantages of inoculation to prevent it*. The text was only published 6 years after the presentation, and years after the publication of d’Alembert’s criticism, which was one of the sources of the conflict.

5 The full title of the table is *An estimate of the degrees of the mortality of mankind, drawn from curious tables of the births and funerals at the city of Breslaw, with an attempt to a scertain the price of annuities upon lives* (Bacaër, 2011, p. 6).

6 Halley’s table starts with 1000 one-year-old children, Bernoulli added a first line of 1300 newborn, explaining in detail the choice of this number.

7 For a more detailed description of the calculations in English, see Colombo and Diamanti (2015) and Bacaër (2011).

8 “Je souhaite seulement que dans une question qui regarde de si près le bien de l’humanité, on ne décide rien qu’avec toute la connaissance de cause qu’un peu d’analyse et de calcul peut fournir” (Bernoulli, 1766, pp. 36–37). Translation from Bacaër (2011, p. 27).

9 [*Mathematical booklets*]. The full title of the study is *Onzième Mémoire: sur l’application du calcul des probabilités à l’inoculation de la petite vérole; notes sur le mémoire précédent; théorie mathématique de l’inoculation* [*Eleventh Memoir: on the application of probability calculus to smallpox inoculation; notes on th eprevious memoir; mathematical theory of inoculation*]*.* An extended version of d’Alembert’s work is published in 1767 under the title of “Réflexions philosophiques et mathématiques sur l’application du calcul des probabilités à l’inoculation de la petite vérole” [Philosophical and mathematical reflections on the application of the calculus of probabilities to smallpox inoculation] in (d’Alembert, 1767).

10 “Je suppose que la vie moyenne d’un homme de trente ans, soit trente autre années, c’est-à-dire, que, suivant les tables de mortalité connues, il puisse raisonnablement espérer de vivre encore trente ans, en s’abandonnant à la nature, et en ne faisant point inoculer. Je suppose ensuite, qu’en se soumettant à cette opération, sa vie moyenne soit de 34 ans, c’est-à-dire, de quatre ans de plus que s’il attendoit la petite Vérole. Je suppose enfin, avec M. Bernoulli, que le risque de mourir de l’inoculation soit de 1 sur 200. Cela posé, il me semble que pour apprétier l’avantage de l’inoculation, il faut comparer, non la vie moyenne de 34 ans à la vie moyenne de trente ; mais le risque de 1 sur 200, auquel on s’expose de mourir en un mois par l’inoculation (et cela à l’âge de trente ans, dans la force de la santé et de la jeunesse), à l’avantage éloigné de vivre quatre ans de plus au bout de soixante ans, lorsqu’on sera beaucoup moins en état de jouir de la vie.” (d’Alembert, 1761, pp. 32–33)

11 “On a trop confondu l’intérêt que l’Etat en général peut avoir à l’Inoculation, avec celui que les particuliers y peuvent trouver; or ces deux intérêts peuvent être fort différents.” (d’Alembert, 1761, p. 37)

12 “Dès qu’on accordera qu’on peut mourir de l’Inoculation, je n’oserai plus blâmer un père qui craindra de faire inoculer son fils. Car si ce fils par malheur en est la victime, son père aura éternellement à se faire le reproche affreux d’avoir avancé la mort de ce qu’il avoit de plus cher; & je ne connois rien à mettre dans la balance vis-à-vis d’un pareil malheur, fait pour répandre sur les jours de ce père infortuné la plus cruelle amertume.” (d’Alembert, 1767, pp. 355–356)

13 “On a tant écrit depuis quelques années pour et contre l’inoculation que le public doit être aujourd’hui plus que suffisamment instruit sur ce sujet et par conséquent fatigué d’avance de tout ce qu’on pourrait ajouter encore, pour éclaircir ou pour embrouiller la question.” (d’Alembert, 1761, p. 26)

14 Especially the works of Clairaut.
